# End-to-End Deep Learning Fusion of Fingerprint and Electrocardiogram Signals for Presentation Attack Detection

**DOI:** 10.3390/s20072085

**Published:** 2020-04-07

**Authors:** Rami M. Jomaa, Hassan Mathkour, Yakoub Bazi, Md Saiful Islam

**Affiliations:** 1 Computer Science Department, College of Computer and Information Sciences, King Saud University, Riyadh 11543, Saudi Arabia; mathkour@ksu.edu.sa (H.M.); saislam@ksu.edu.sa (M.S.I.); 2 Computer Engineering Department, College of Computer and Information Sciences, King Saud University, Riyadh 11543, Saudi Arabia; ybazi@ksu.edu.sa

**Keywords:** fingerprint, ECG, presentation attack detection, deep learning

## Abstract

Although fingerprint-based systems are the commonly used biometric systems, they suffer from a critical vulnerability to a presentation attack (PA). Therefore, several approaches based on a fingerprint biometrics have been developed to increase the robustness against a PA. We propose an alternative approach based on the combination of fingerprint and electrocardiogram (ECG) signals. An ECG signal has advantageous characteristics that prevent the replication. Combining a fingerprint with an ECG signal is a potentially interesting solution to reduce the impact of PAs in biometric systems. We also propose a novel end-to-end deep learning-based fusion neural architecture between a fingerprint and an ECG signal to improve PA detection in fingerprint biometrics. Our model uses state-of-the-art EfficientNets for generating a fingerprint feature representation. For the ECG, we investigate three different architectures based on fully-connected layers (FC), a 1D-convolutional neural network (1D-CNN), and a 2D-convolutional neural network (2D-CNN). The 2D-CNN converts the ECG signals into an image and uses inverted Mobilenet-v2 layers for feature generation. We evaluated the method on a multimodal dataset, that is, a customized fusion of the LivDet 2015 fingerprint dataset and ECG data from real subjects. Experimental results reveal that this architecture yields a better average classification accuracy compared to a single fingerprint modality.

## 1. Introduction

**Figure 1:**
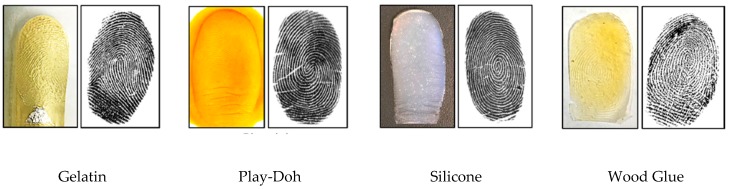
Examples of fingerprint artefacts fabricated using different materials. A real image of a fabricated fingerprint is shown on the left and a scanned image using a fingerprint sensor is shown on the right [4].

Biometric systems in which the physiological or behavioral characteristics of humans, e.g., fingerprints, electrocardiogram (ECG), gait, iris, and face, are captured and utilized for authentication are increasingly used. Fingerprints are among the most extensively employed biometrics owing to their several advantages, such as acceptability, collectability, and high authentication accuracy [1]. The widespread availability of fingerprint-based systems has made them vulnerable to numerous attacks, mainly presentation attacks (PAs). ISO/IEC 30107 defines a PA as the presentation of a fraudulent sample, such as an artefact or a fake biological sample, to an input biometric sensor with the intention of circumventing the system policy [2]. An artefact can be an artificial or synthetic fingerprint presented as a copy of a real fingerprint, which is also known as a spoof [3]. Figure 1 shows examples of numerous artefact fingerprint samples created by different artificial materials, such as gelatin, Play-Doh, and silicon [4]. For fake biological sample-based attacks, a severed or altered finger, or a finger of a cadaver, is presented to deceive a biometric sensor. The automated process used for detecting a PA in a biometric system is called PA detection (PAD) [2]. The aim of PAD is discriminating the bona fide (i.e., real or live) biometric samples from PA (i.e., artefact) samples. 

Fingerprint PAD methods can be divided into hardware- and software-based methods [5]. In a hardware-based method, additional hardware devices are added to the biometric system to capture additional characteristics indicating the liveness of the fingerprint, such as blood pressure in the fingers, skin transformation, and skin odor [6,7,8]. With software-based methods, in contrast, the PAs of the fingerprints are analyzed by applying image processing techniques on fingerprint images. By exploring software-based techniques for fingerprint PAD studied in the literature, these methods can be grouped into handcrafted feature- and deep-learning-based techniques. In handcrafted feature-based techniques, expert knowledge is required to formulate the feature descriptors, whereas in deep-learning-based techniques, no such expert knowledge is required.

The local binary pattern (LBP) is one of the earliest and most common handcrafted techniques that has been investigated for fingerprint liveness detection, in which LBP histograms are applied to extract the texture liveness information using binary coding [9]. Measuring the loss of information while fabricating fake fingerprints is utilized in local phase quantization (LPQ) to differentiate between bona fide and artefact fingerprint images [10]. The Weber local descriptor (WLD) is applied for fingerprint liveness detection, in which 2D histograms representing differential excitation and orientation features are applied [11]. Combining these local descriptors such as WLD with LPQ [11], or WLD with LBP [12], improves the accuracy of detecting the liveness of a fingerprint. A new local contrast phase descriptor is proposed for fingerprint liveness detection as 2D histogram features composed of spatial and phase information [13].

Deep learning techniques have recently proven their superiority over traditional approaches in image classification problems [14,15]. Deep learning techniques have also proven their advantages on 1D signals, including ECG [16,17,18,19]. Several studies have investigated the utilization of deep learning techniques in biometrics systems [20,21,22], and for fingerprint PAD [23,24,25,26]. Convolutional neural network (CNN) networks have exhibited continuous improvements for spoof detection compared with handcrafted techniques. An early work that introduced CNN for fingerprint PAD [23] employed transfer learning using a pre-trained CNN model for detecting fake fingerprints, which achieved the best results in the LivDet 2015 competition [27]. Another use of deep learning for fingerprint PAD is presented in [28], where local patches of minutiae have been extracted and processed using a well-known CNN model called Inception-V3, which achieved state-of-the-art accuracy in fingerprint liveness detection. A CNN model with improved residual blocks was proposed to balance between the accuracy and the convergence time in a fingerprint liveness system [29], wherein they extracted local patches using the statistical histogram and center of gravity. This approach won first place in the LivDet 2017 competition. A small CNN network was proposed to overcome the difficulties in the deployment of a fingerprint liveness detection system in mobile systems by utilizing the structure of the SqueezNet fire module and removing the fully connected layers [24].

Recently, a new group of fingerprint PAD methods have also been considered, which fall outside of software- and hardware-based approaches and are based on the fusion of fingerprint with a more secure biometric modality [30,31]. Several researchers investigated the fusion of fingerprints with a variety of biometric modalities, such as face, ECG, and fingerprint dynamics, to improve the accuracy and security of biometric systems [19,32,33,34,35,36,37,38,39,40,41]. The fusion of an ECG with other biometric modalities [37,38,42,43,44,45,46] has also received attention because the ECG has certain biometric advantages, such as a natural inherence of the liveness characteristic and a continuous authentication over time [47]. The crucial location of the heart in the body enables this biometric to be used as a secure modality. Moreover, a high-quality ECG can be captured from fingers, which make this modality a convenient candidate for a multimodal fusion with fingerprints [48]. These characteristics render ECG biometrics robust against PAs and provide them with advantages over other traditional biometrics. Several studies have considered the fusion of fingerprints and an ECG for PAD in fingerprint biometrics. A sequential score level fusion between an ECG and a fingerprint was proposed in [37]. Later, this approach was improved to be appropriate for fingerprint PAD in an authentication system [38]. Another study on fusing a fingerprint with an ECG was proposed in [36], in which the fusion is achieved at the score level by applying an automatic updating of the ECG templates. In this study, the authors fused an ECG matching score with the liveness score to evaluate the liveness of the fingerprint sample, demonstrating a good performance.

Several recent studies have proposed utilizing a CNN to deal with two-branch networks for processing video data [49,50,51]. A CNN network has been introduced into a multimodal biometric system combining an ECG with a fingerprint [19,52], in which the CNN is used for extracting ECG and fingerprint features. Although CNN was used in these studies, they did not achieve an end-to-end fusion in which the CNN is only used as features extractor and the classification carried out by an independent classifier. Furthermore, these studies focused on authentication performance rather than fingerprint PAD.

In this study, we propose a novel architecture for fusing a fingerprint and an ECG to detect and prevent fingerprint PAs. The proposed architecture is learnable end-to-end from the signal level to the final decision. The proposed method is intended to achieve a high degree of robustness against the PA targeting of a fingerprint modality. We evaluated the proposed system using a customized dataset composed of fingerprints and ECG signals. 

The main contributions of this paper are listed as follows:

- Proposal of a novel end-to-end neural fusion architecture for fingerprints and ECG signals.
- A novel application of state-of-the-art EfficientNets for fingerprint PAD.
- Proposal of a 2D-convolutional neural network (2D-CNN) architecture for converting 1D ECG features into 2D images, yielding a better representation for ECG features compared to standard models based on fully-connected layers (FC) and 1D-convolutional neural networks (1D-CNNs).

The remainder of this paper is organized as follows. In Section 2, we introduce our proposed end-to-end deep learning approaches. In Section 3, we present the datasets and experimental setup applied. In Section 4, we present experimental results and discussions. Finally, in Section 5, we provide some concluding remarks and suggest areas of future study.

## 2. Proposed Methodology

Assume a fingerprint dataset $D={\left\{X_{i},\;y_{i}\right\}}_{i=1}^{N}$ composed of N=A+B (where A is the number of artefact samples and B is the number of bona fide samples), where $X_{i}$ represents the input fingerprint image and $y_{i}$ is a binary label indicating if a fingerprint is an artefact or a bona fide (real). The aim of ordinary fingerprint PAD is to detect whether a fingerprint image is a PA (artefact) and differentiate it from a bona fide fingerprint sample. In this study, we consider ECG signals as an additional input modality to strengthen the fingerprint PAD system. To this end, the dataset becomes triplet $D={\left\{\left(X_{i}^{f},X_{i}^{e}\right),\;y_{i}\right\}}_{i=1}^{N}$, where $X_{i}^{f}$ is the fingerprint image and $X_{i}^{e}$ is the ECG signal. 

**Figure 2:**
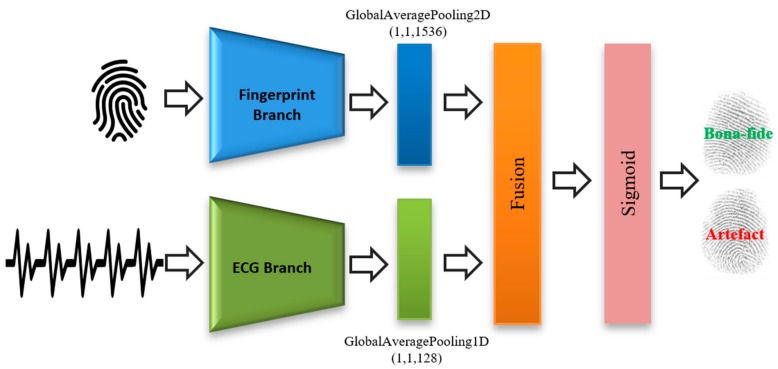
Overall architecture of the proposed end-to-end convolutional neural network-based (CNN) fusion architecture. ECG, electrocardiogram.

Figure 2 shows the proposed fusion approach, which is composed of three parts, i.e., the fingerprint branch, the ECG branch, and a fusion module. Detailed descriptions for these branches are provided in the next subsections.

### 2.1. Fingerprint Branch

**Figure 3:**
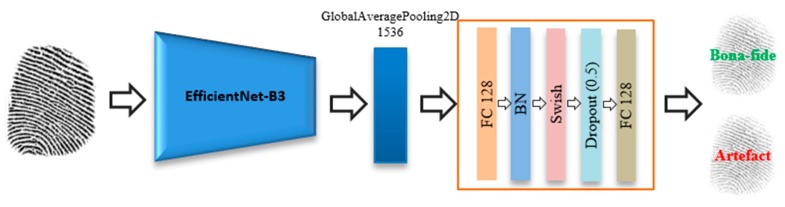
Flowchart of a fingerprint branch.

A fingerprint branch uses state-of-the-art EfficientNets [53] to obtain the feature representations of a fingerprint as shown in Figure 3 EfficientNets are a family of models that were recently developed by the Google Brain team by applying a new model scaling method for balancing the depth, width, and resolution of the CNNs [53]. Their scaling method uniformly scales the dimensions of a network using a simple and efficient compound coefficient. The compound scaling method enables a baseline CNN network to be scaled up with respect to the available resources while maintaining a high efficiency and accuracy. EfficientNets include mobile inverted bottleneck convolution (MBConv) as the basic building block [54]. In addition, this network uses an attention mechanism based on squeeze excitation (SE) to improve feature representations. This attention layer starts by applying a global average pooling (GAP) after each block. This operation is then followed by a fully-connected layer (with weight $W_{1}$) to reduce the number of dimensions by (1/16). The resulting feature vector s is then used to calibrate the feature maps of each channel (V) using a channel-wise scale operation with an extra fully-connected layer with weight $W_{2}$. SE operates as shown below:

(1) $$s=Sigmoid\left(W_{2}\left(ReLU\left(W_{1}\left(V\right)\right)\right)\right),$$

(2) $$V_{SE}=s⊙V,$$

where s is the scaling factor, ⊙ refers to the channel-wise multiplication, and V represents the feature maps obtained from a particular layer of the EfficientNet. 

**Figure 4:**
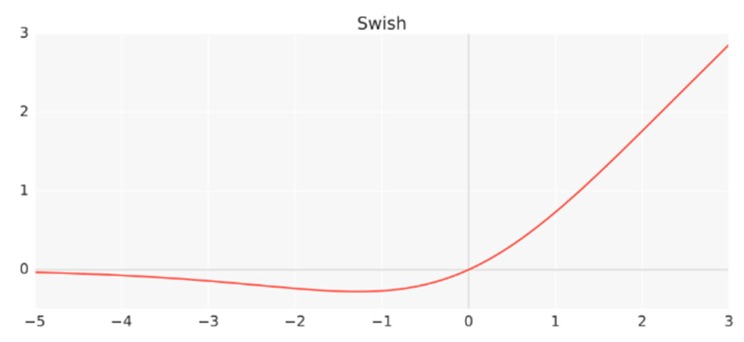
Swish activation function [55].

Furthermore, a novel activation function called Swish is used by an EfficientNet, which is essentially the sigmoid function multiplied by x according to Equation (3). Figure 4 shows the behavior of the following Swish activation function:

(3) f(x)=x·Sigmoid(x)

**Figure 5:**
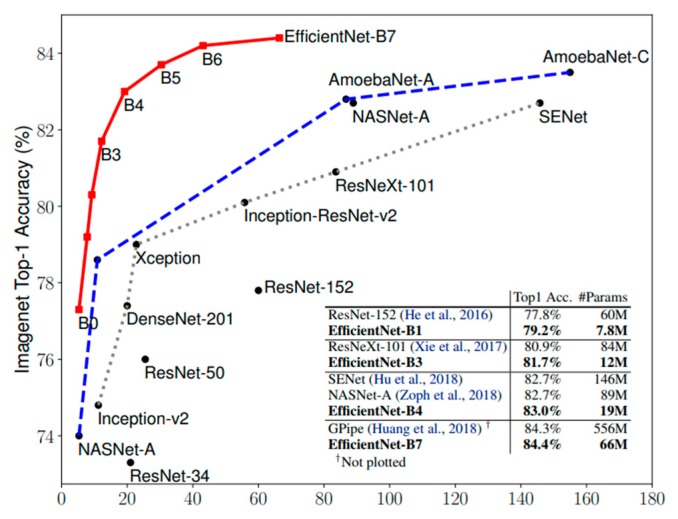
Comparison among EfficientNet and other popular CNN models in terms of ImageNet accuracy vs. model size [53].

EfficientNet models surpass the accuracy of state-of-the-art CNN approaches on the ImageNet dataset [56] by minimizing the numbers of parameters and FLOPS, as shown in Figure 5. In this study, we investigate the baseline EfficientNet-B3 in terms of the feature representations of a fingerprint. To the best of our knowledge, this is the first time EfficientNets have been used for fingerprint PAD. 

During the experiments, we truncated EfficientNet-B3 by removing its 1000 softmax classification layer and used the output of the “swish_78” layer as an input to a fusion module, which has the task of fusing the fingerprint and ECG features, as described later.

### 2.2. ECG Branch

**Figure 6:**
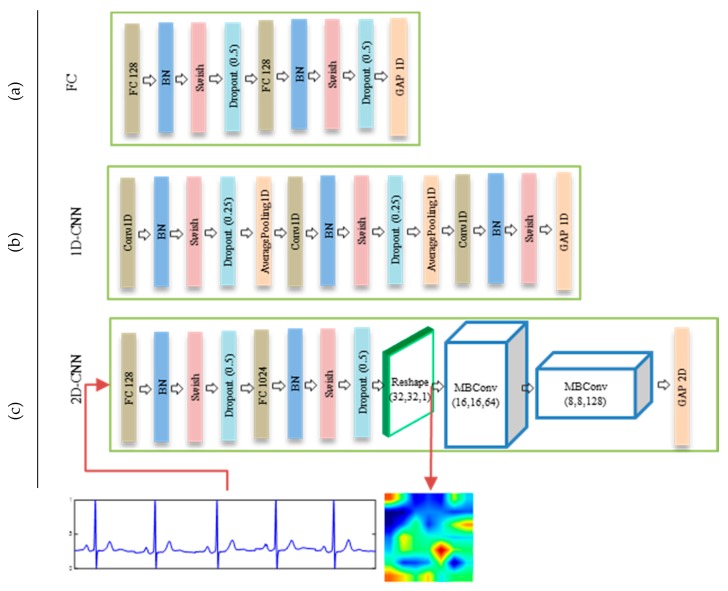
Details of ECG feature extraction architectures in ECG branch: (**a**) FC (**b**) 1D-CNN, and (**c**) 2D-CNN.

Regarding an ECG branch, we propose three different feature representation architectures, FC, 1D-CNN, and 2D-CNN, as shown in Figure 6. The FC architecture is composed of simple fully-connected layers followed by batch normalization (BN), a Swish activation function, and dropout regularization to reduce an overfitting, as presented in Figure 6a. The second architecture, 1D-CNN, is based on the application of 1D convolution operations on the ECG signals. With this architecture, the ECG signals are fed through three 1D consecutive convolutional layers, the first two layers of which are followed by a BN, Swish, dropout (0.25), and 1D average pooling, and the last layer is followed by a BN, Swish, and 1D average pooling, as shown in Figure 6b. The last architecture, which is one of the main contributions of the present paper, is based on the idea of converting ECG feature into a 2D features image using a generator module and then processing the signal using a standard 2D CNN network, as shown in Figure 6c. In particular, this architecture learns and reshapes a 1D ECG feature into a 2D image using fully connected layers. The resulting image is then fed to two consecutives MBConv blocks to obtain the final 2D representations. Transforming a 1D ECG feature into an image can play a significant role in achieving powerful 2D convolution and pooling operations when learning the appropriate ECG features. During the experiments, we show that this architecture allows the generation of a better representation compared to those based on an analysis of 1D features. 

### 2.3. Fusion Module 

**Figure 7:**
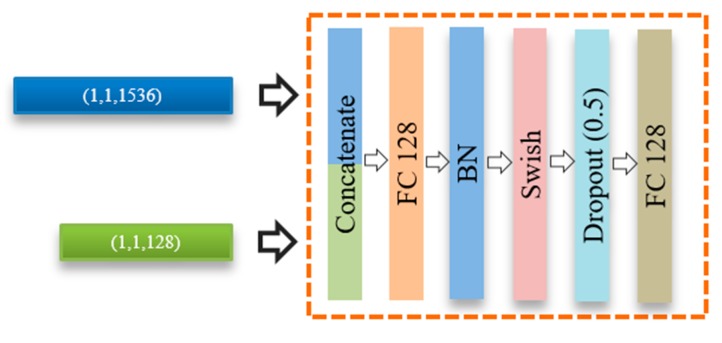
Structure of the fusion module.

The feature representations obtained from both fingerprint and ECG branches are further processed using a fusion module. This fusion module is composed of a sequence of layers, as shown in Figure 7. First, the feature vector of an ECG is concatenated into a fingerprint feature vector to produce a single global feature vector. The concatenated feature is fed to an additional fully connected layer followed by a BN, Swish activation function, and dropout (0.5) regularization. Finally, the output of this module is fed to a binary classification layer using a sigmoid activation function to determine the final fingerprint class, i.e., an artefact or bona fide. 

### 2.4. Network Optimization

As mentioned previously, the complete architecture proposed in this study is a learnable end-to-end network using a backpropagation algorithm. If we define the output of the sigmoid function in the final layer of the trained network as ${\hat{y}}_{i}$, then the distribution of the network output ${\hat{y}}_{i}$ follows a Bernoulli distribution. The determination of the weights W of the network, including those of the fingerprint and ECG branches, can be carried out by maximizing the following likelihood function:

(4) $$L\left(D,W\right)=\prod_{i=1}^{N}{\hat{y}}_{i}{}^{y_{i}}{\left(1-{\hat{y}}_{i}\right)}^{1-y_{i}},$$

which is equivalent to minimizing the following log-likelihood function:

(5) $$L\left(D.W\right)=-\sum_{i=1}^{N}y_{i}ln\left({\hat{y}}_{i}\right)+\left(1-y_{i}\right)ln\left(1-{\hat{y}}_{i}\right).$$

The loss function in (5) is usually called a cross-entropy loss function. To optimize this loss, we use the RMSProp optimization algorithm proposed by Hinton [57], which is considered one of the most common adaptive gradient algorithms, dividing the gradient by averaging the magnitude of its recent movement as follows:

(6) $$E{\left[g^{2}\right]}_{t}=\beta E{\left[g^{2}\right]}_{t-1}+\left(1-\beta \right){\left(\frac{\partial L}{\partial W}\right)}^{2},$$

(7) $$W_{t}=W_{t-1}-\alpha \left(\frac{\partial L}{\partial W}\right)\frac{1}{\sqrt{E{\left[g^{2}\right]}_{t}}},$$

where $E{\left[g^{2}\right]}_{t}$ represents a moving average of the squared gradients during the iteration process (t), and $\frac{\partial L}{\partial W}$ is known as the gradients of the loss function of the weights of the network W. Parameters α and β are the learning rate and moving average, respectively. During the experiments, parameter β is set to its default value (β=0.9), whereas α is initially set to 0.0001 and is periodically decreased by a factor of 1/10 for every 20 epochs. 

## 3. Experiments 

### 3.1. Datasets

**Figure 8:**
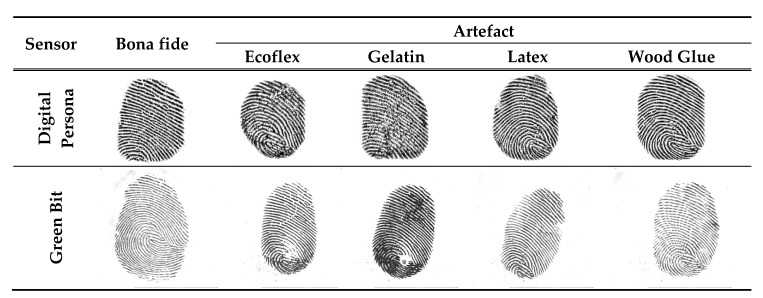
Bona fide and artefact fingerprint samples from the LivDet 2015 dataset captured using Digital Person and Green Bit sensors. Artefact samples were fabricated using different materials.

To evaluate the proposed approach, we used the LivDet 2015 dataset for the fingerprint and real ECG datasets collected in our lab. LivDet 2015 has approximately 19,000 images divided into two parts: training and testing. Each part has bona fide (live) and artefact (fake) images captured using different fingerprint sensors, as shown in Table 1. Numerous materials are used for fabricating the artefact fingerprint samples, e.g., Ecoflex, gelatin, latex, and wood glue. The testing dataset contains artefact samples fabricated using various materials, some of which are not used in the training dataset, such as OOMOO and RTV, as shown in Table 2. Figure 8 shows bona fide and artefact samples for the same subject captured from two different sensors, i.e., Green Bit and Digital Persona sensors.

**Figure 9:**
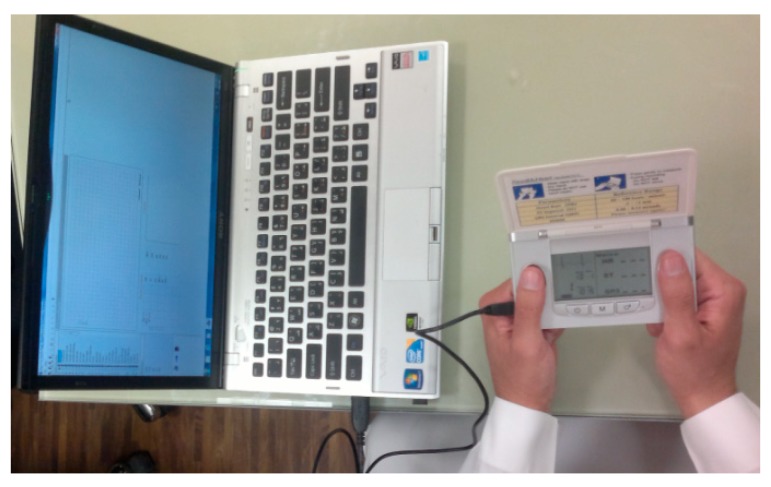
ECG data collection using the ReadMyHeart device.

**Figure 10:**
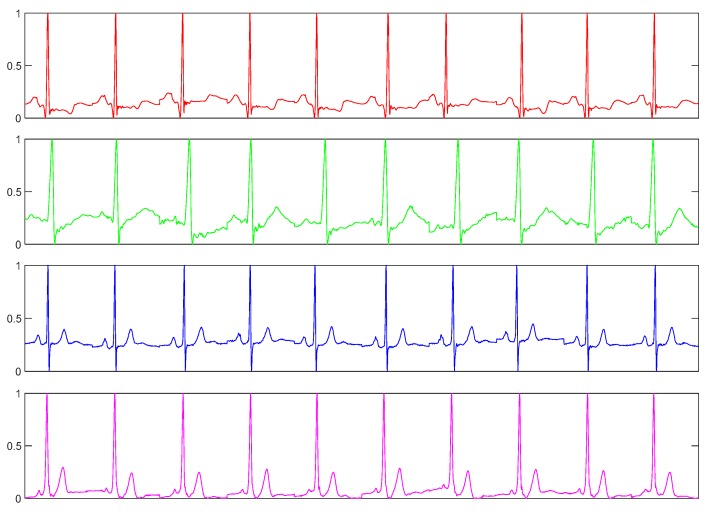
ECG sample of 10 heart beats from four different subjects.

For the ECG dataset, we used a dataset collected in our lab. We collected this dataset using a commercially available handheld ECG device, i.e., ReadMyHeart by DailyCare BioMedical, Inc. (https://www.dcbiomed.com/webls-en-us/index.html), as shown in Figure 9. We built a database of 656 ECG records captured from 164 individuals collected in two sessions [48,58]. Now, we have extended this database with a third session to have 10 records for most of the users. The device captures a signal for 15 seconds, digitalizes it and exports it to the computer as an ECG record. Generally, such a signal may contain different types of noise, such as power-line interface, baseline wanders, and patient-electrode motion artifacts. In the preprocessing step, we use a band-pass Butterworth filter of order four with cut-off frequencies of 0.25 and 40 Hz to remove the noises. Then an efficient curvature-based method is used to detect heartbeats [59,60] and we take the first 10 beats from each record for this experiment. Figure 10 shows such preprocessed ECG samples from four different subjects.

Owing to a lack of availability of public multimodal datasets containing fingerprint and ECG signals, we constructed a multi-model dataset from the LivDet 2015 dataset and an ECG dataset. First, we built a mini fingerprint dataset from the LivDet 2015 dataset, called the mini-livdet2015 dataset, containing images from Digital Persona sensor. This mini-livdet2015 is composed of 70 subjects, each of which has bona fide and artefact samples (10 and 12, respectively). Subsequently, we randomly selected the artefact samples from all available fabricating materials. To form this multimodal dataset, we assigned a random subject from the ECG dataset to each subject from the mini-livdet2015 dataset. Table 3 describes this new dataset, which is comprised of 70 subjects, each of which has 10 bona fide and 12 artefact fingerprint samples, and 10 ECG samples. 

During training, we feed the network with batches of input triplets that cover both possible classes. For the bona fide label, we assign a bona fide fingerprint sample with a bona fide ECG sample from the same subject; i.e., $X_{i}^{f}$ and $X_{i}^{e}$ are bona fide samples belonging to the same subject. Because we do not have artefact ECG signals, we assign an artefact fingerprint sample from one subject with a bona fide ECG sample from another subject (zero-effort ECG sample); i.e., $X_{i}^{f}$ and $X_{i}^{e}$ are bona fide samples from two different subjects. 

Feeding the network with these inputs allows learning the correlations between bona fide fingerprint samples and ECG samples of the same subject to correctly predict which samples are bona fide. Furthermore, this network learns how to correctly predict an artefact by learning the features representing the incoherence between artefact fingerprint sample and a bona fide ECG sample of the same subjects or between a bona fide fingerprint sample of a subject and a bona fide ECG sample belonging to different subjects.

### 3.2. Experiment Setup

To evaluate the proposed approach, we conducted several experiments. First, we carried out an initial experiment to evaluate the performance of the fingerprint branch net regarding the detection of PAs. We compared our results with previous state-of-the-art methods. For this purpose, we utilized the LivDet 2015 dataset, whereas the fingerprint branch net was trained on the training portion of the LivDet 2015 and tested on the testing portion of the same dataset. In the second experiment, we evaluated the three proposed fusion architectures in detecting and preventing the PAs. We then conducted an experiment to analyze the sensitivity of the highest performing architecture during the second experiment. To this end, we analyzed the effects of increasing the number of subjects during the training on the classification accuracy. Finally, we reported the number of parameters and classification time by the proposed architectures compared with state-of-the-art methods. 

For training the network, we use the RMSProp optimizer with the following parameters: β is set to its default value (β=0.9), whereas α is initially set to 0.0001 and is periodically decreased by a factor of 1/10 for every 20 iterations (epochs). For compatibility with the LivDet 2015 competition [27], the accuracy was used as the evaluation parameter in all of the experiments. The accuracy is defined as the percentage of correctly classified samples. 

All experiments were repeated five times and the average classification accuracy was reported. The experiments were carried out using a workstation with i9 CPU @ 2.9 GHz, 32 GB of RAM, and NVIDIA GeForce GTX 1080 Ti (11 GB GDDR5X). 

## 4. Results and Discussions

### 4.1. Experiments Using Fingerprint Modality Only

Initially, we examined the performance of the proposed fingerprint network based on EfficientNet. This evaluation allows us to compare the performances of this network in terms of fingerprint PAD with that of the methods proposed in the LivDet 2015 competition [27]. Table 4 shows the results after training the network for 50 iterations.

**Figure 11:**
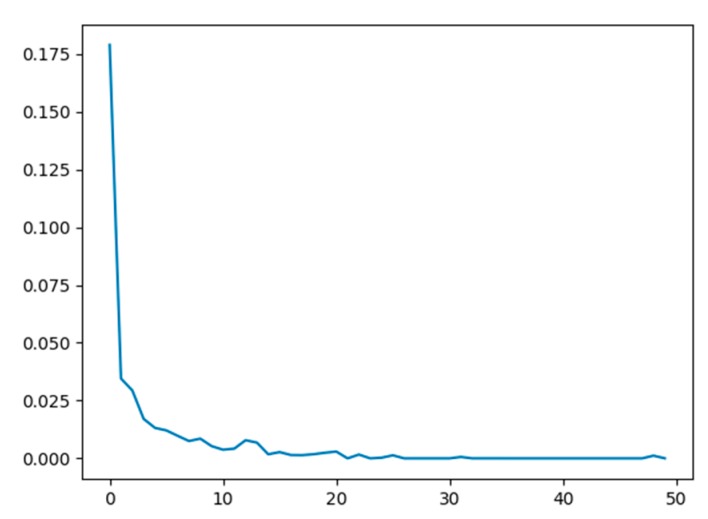
Model loss versus number of epochs (50) by training on LivDet 2015 dataset.

We can see from the results in Table 4 that the proposed fingerprint network achieves an overall classification accuracy of 94.87%. A comparison of the reported accuracy of the proposed network with those reported from the LivDet 2015 competition shows that our method would have been the second-best approach. Moreover, the proposed method follows the same behavior as the other two algorithms in terms of its accuracy for the individual sensors, achieving a high accuracy of 97.29% for the Crossmatch sensor (i.e., an easy to learn sensor) and a relatively lower accuracy of 91.96% for the Digital Persona sensor (i.e., a difficult to learn sensor). Furthermore, the proposed method achieves moderately high accuracies of 94.68% and 95.12% for the Green bit and Biometrika sensors, respectively. Figure 11 shows the progress of the loss function during the training on the LivDet 2015 dataset (training part). Note that the loss converges at a low number of iterations (nearly 25 iterations). The reported results confirm the promising capability of the network in detecting PAs, motivating us to improve it further by proposing a multimodal solution that fuses fingerprints with ECG signals.

### 4.2. Fusion of Fingerprints and ECGs

As mentioned previously, owing to the lack of a multimodal dataset containing fingerprints and ECG modalities, we built a mini-livdet2015 dataset and fused it with the ECG dataset. We used a Digital Persona sensor, the most difficult sensor used for the LivDet 2015 dataset, as demonstrated in the previous experiment, and achieved the lowest accuracy 91.96% in comparison to the other sensors. This mini-livdet2015 contains 70 subjects, each of which has 10 bona fide and 12 artefact fingerprint samples. We constructed the multimodal dataset by randomly linking each subject from the mini-livdet2015 dataset to a subject from the ECG dataset, as previously discussed. Before running the fusion network on this multimodal dataset, we first trained the fingerprint network on the mini-livdet2015 dataset to obtain an indication regarding its performance, which is considered a baseline for our fusion mechanism. We obtained an accuracy of 92.98% using 50% of the subjects for training and 50% for testing, i.e., 35 subjects for training and the other 35 subjects for testing.

After this step, we evaluated the complete architecture using the three proposed feature extraction solutions (i.e., FC, 1D-CNN, and 2D-CNN). Table 5 shows the average classification accuracy of the three fusion architectures. Furthermore, the average classification accuracy of the fingerprint network on the mini-livdet2015 dataset is reported. 

The reported results show that fusing fingerprints with ECG data clearly improves the accuracy of artefact fingerprint detection. The different architectures, namely, FC, 1D-CNN, and 2D-CNN, achieve accuracies of 94.99%, 94.84%, and 95.32%, respectively, thereby outperforming the accuracy achieved by fingerprint net (i.e., without applying a fusion). As the results indicate, the 2D-CNN model achieves the highest accuracy (95.32%) compared with the other two fusion architectures. The high performance of the 2D-CNN model can be attributed to the conversion of ECG signals into images, thus utilizing the power of 2D convolution and pooling operations, in addition to the introduction of MBConv blocks as the main blocks for learning the representative features. 

### 4.3. Sensitivity Analysis of the Number of Training Subjects

During this experiment, we discuss how the number of subjects used for training can affect the level of accuracy. We repeated the above experiment with different percentages of subjects used for training (between 20% and 80%), the average accuracy of which is reported in Table 6. 

The reported results show that increasing the number of subjects (70% and 80%) during the training improves the testing accuracy. Although this behavior is the same for the three proposed architectures, we can see that the 2D-CNN model consistently outperforms the other two models with an accuracy of 97.10% when using 80% of the subjects in the dataset for training. In contrast, decreasing the number of subjects during the training degrades the testing accuracy. Despite the decrease in testing accuracy, the level achieved is still acceptable (89.71%, 89.31%, and 90.79%) for the FC, 1D-CNN, and 2D-CNN, respectively when using 20% of the subjects for training. 

### 4.4. Sensitivity with Respect to the Pre-Trained CNN

In order to further assess the sensitivity of the proposed approach with respect to the pre-trained CNN model, we carried out additional experiments using other well-known pre-trained CNN models: Inception-v3 [61], DenseNet [62], and residual network (ResNet) [63]. We used these recent pre-trained models for this experiment as they require a comparatively small number of parameters as shown in Table 7. 

Inception-v3 is one of Inception models family developed by Google [61], in which they introduced the concept of factorizing of convolutions. Inceptions models are based on increasing the width and depth of the network, by utilizing a module called inception [64], which contains several convolutional layers with different filter sizes. The utilization of the inception module allows the Inception model for a better deal with scale and spatial variations. DenseNet was proposed by Szegedy et al. [62] for better utilization of computing resources. DeneNet is based on adding connections between each layer and every other layer in feed-forward fashion, in which each layer receives the feature maps of all preceding layers as input and feeds its own feature maps as input into all subsequent layers. ResNet was proposed by He et al. [63] to overcome the difficulties in training deeper networks by learning residual functions. These residual networks achieved better optimization and generalization as the depth increases.

We note from the results in Table 7, that EfficientNet-B3 achieves the highest accuracies in the three architectures and outperforms the other pre-trained CNNs. The accuracies of EfficientNet-B3 in the three architecture (94.99%, 94.84%, and 95.32%) consistently exceed the best accuracies of the other models except for Inception-v3 which achieves a comparable result in the case of 2D-CNN (95.20%). However, EfficientNet-B3 requires the minimum number of parameters (10 M after removing the top layers), whereas Inception-v3 and resNet-50 require 21 M and 23 M, respectively (after removing the top layers). Finally, from the reported accuracies, we note that 2D-CNN outperforms the FC and 1D-CNN for all the pre-trained models (i.e., 95.32%, 95.20%, 93.29%, and 94.00% for EffieicentNet-B3, Inception-v3, DenseNet-169, and ResNet-50 respectively). 

### 4.5. Sensitivity of the ECG Network Architecture 

In order to further assess the sensitivity of the proposed approach with different configurations, we carried out additional experiments to show the effect of using different configurations of the ECG branch net on the overall accuracy. Considering that the 2D-CNN architecture proves its superiority over the FC and 1D-CNN as shown in the previous sections, we reported the experiments that cover applying different configurations using 2D-CNN architecture. We tested 8 different configurations as described in Table 8. Let us consider the configuration #8, which is shown in Figure 6c: (2 fc = (128, 1024), 2 blocks MBConv (64, 128), fc = 128), this means we use two consecutive fully-connected layers of size 128 and 1024, respectively, in addition to using two consecutive MBConv blocks, of depth 64 and 128, respectively, and finally one fully-connected layer of size 128. The second fully-connected layer fc (1024) means that the feature vector is reshaped into (32 × 32 × 1) as shown in Figure 6c. 

From the reported results in Table 8, we note the following points. Removing the first fully-connected layer fc (128) in configuration #1, degraded the accuracy (91.90%), whereas increasing the feature map in configuration #2 by replacing the second fully-connected layer fc (1024) with fc (4096); i.e., the feature vector is reshaped into (64 × 64 × 1); will not significantly improve the accuracy (93.56%). Furthermore, changing the number and sizes of MBConv blocks up to 3 (configurations #6 & #7) or down to 1 (configurations #3, #4, & #5), produces better accuracies up to 95.56% in configuration #4. In the proposed configuration #8, we used 2 MBConv blocks, in which the networks achieved the second best accuracy of 95.32%. 

### 4.6. Classification Time 

In this study, we used an EfficientNet with 12 million parameters as the main building block for the fingerprint branch. This model provides impressive results with a low computational cost. In particular, our models converge using only 50 epochs. The complete architecture provides an average classification time for one subject (i.e., fingerprint image + ECG signal) of 30–35 ms (depending on the architecture), which is faster than previous state-of-the-art approaches (i.e., 128 ms [24] and 800 ms [28]). Recall that the approaches described in [21] and [24] applied solutions using only the fingerprint modality and networks with a larger number of weights. 

## 5. Conclusions

In this paper, we proposed an end-to-end deep learning approach fusing fingerprint and ECG signals for boosting the PAD capabilities of fingerprint biometrics. We also introduced EfficientNet, a state-of-the-art network, for learning efficient fingerprint feature representations. The experimental results prove the superiority of the EfficientNet over other known pre-trained CNNs in terms of accuracy and efficiency. For the ECG signals, we proposed three different architectures and configurations FC, a 1D-CNN, and a 2D-CNN. With the 2D-CNN model, we transformed the ECG features into images using a generator network. The experimental results obtained on a multimodal dataset composed of fingerprint and ECG signals reveal the promising capability of the proposed solution in terms of the classification accuracy and computation time. Although we used a customized database of fingerprint and ECG signal to validate the proposed method, we intend to use a database of fingerprint and ECG signal captured simultaneously using a multimodal sensor. Since this type of sensor is not commercially available, we would like to develop a prototype of such a sensor, create a database of real multimodal data, and use it for the validation of the proposed method as our future work.

## Figures and Tables

**Table 1:** Device and image characteristics of the LivDet 2015 dataset.

Sensor	Resolution (dpi)	Image Size (pixel)	Training		Testing	
			Live	Fake	Live	Fake
**Green Bit**	**500**	500 × 500	1000	1000	1000	1500
**Biometrika (Hi Scan)**	1000	1000 × 1000	1000	1000	1000	1500
**Digital Persona**	500	252 × 324	1000	1000	1000	1500
**Crossmatch**	500	640 × 480	1500	1500	1500	1448

**Table 2:** Materials used for fabricating fake images in the LivDet 2015 dataset. Some materials in the testing are unknown during training (underlined).

Sensor	Training	Testing
**Green Bit**	Ecoflex, gelatin, latex, wood glue	Ecoflex, gelatin, latex, wood glue, Liquid Ecoflex, RTV
**Biometrika**		
**Digital Persona**		
**Crossmatch**	Body Double, Ecoflex, PlayDoh	Body Double, Ecoflex, PlayDoh, OOMOO, gelatin

**Table 3:** Description of the customized multimodal dataset, which contains 70 subjects.

	Fingerprint		ECG
	Bona Fide	Arteact	Bona Fide
**Number of samples per subject**	10	12	10
**Total number of samples**	700	840	700

**Table 4:** Comparison between the results of the proposed fingerprint branch net and the best methods from the LivDet 2015 competition, where we present the average accuracy %.

Algorithm	Green Bit	Biometrika	Digital Persona	Crossmatch	Overall
Nogueira (first place winner)	95.40	94.36	**93.72**	**98.10**	**95.51**
**Proposed**	94.68	95.12	91.96	97.29	94.87
Unina (second place winner)	**95.80**	**95.20**	85.44	96.00	93.92

**Table 5:** Average accuracy of three proposed fusion architectures and the fingerprint branch net. The reported results are achieved on the customized dataset.

Biometric Modality	ECG Architecture	Average Accuracy %
Fingerprint	(No fusion)	92.98
Fingerprint + ECG	FC	94.99
	1D-CNN	94.84
	2D-CNN	**95.32**

**Table 6:** Sensitivity analysis of the proposed architectures against the number of training subjects in terms of the reported testing accuracy (ACC %).

ECG Architecture	Percentage of Subjects Used for Training				
	20%	30%	50%	70%	80%
FC	89.71	93.90	94.49	93.92	96.17
1D-CNN	89.31	92.45	94.26	93.36	96.95
2D-CNN	**90.79**	**94.08**	**95.32**	**95.61**	**97.10**

**Table 7:** Classification Accuracy using different pre-trained CNN models. We used Inception-v3, DenseNet-169, and ResNet-50.

CNN Model	Architecture	#Parameters	Average Accuracy %
**EfficientNet-B3**	FC	**10 M**	**94.99**
	1D-CNN		**94.84**
	2D-CNN		**95.32**
Inception-v3	FC	21 M	92.80
	1D-CNN		94.32
	2D-CNN		95.20
DenseNet-169	FC	12 M	91.28
	1D-CNN		92.92
	2D-CNN		93.29
ResNet-50	FC	23 M	93.56
	1D-CNN		93.68
	2D-CNN		94.00

**Table 8:** Classification accuracy of 2D-CNN network by applying three different configurations for ECG architecture.

Configuration	Configuration Description	Accuracy %
1	2 fc = ( 1024), 2 blocks MBConv (64, 168), fc = 128	91.90
2	2 fc = (128, 4096), 2 blocks MBConv (64), fc = 128	93.56
3	2 fc= (128, 1024), 1 block MBConv (32), fc = 128	94.82
4	2 fc = (128, 1024), 1 block MBConv (64), fc = 128	**95.58**
5	2 fc = (128, 1024), 1 block MBConv (128), fc = 128	95.07
6	2 fc = (128, 1024), 3 blocks MBConv (64), fc = 128	94.68
7	2 fc = (128, 1024), 3 blocks MBConv (64, 128, 128), fc = 128	95.20
8 (Proposed)	2 fc = (128, 1024), 2 blocks MBConv (64, 128), fc = 128	95.32
